# Inclusion of Polyphenol-Rich Olive Cake in Beef Cattle Diets: Effects on Meat Quality and Nutritional Traits

**DOI:** 10.3390/ani16050729

**Published:** 2026-02-26

**Authors:** Marianna Oteri, Daniela Beghelli, Vincenzo Lopreiato, Biagio Tuvè, Luigi Liotta, Gianni Dipasquale, Maria Elena Furfaro, Fabiana Antognoni, Mariacaterina Lianza, Vincenzo Chiofalo

**Affiliations:** 1 Department of Veterinary Sciences, University of Messina, Polo Universitario Annunziata, Via Palatucci snc, 98168 Messina, ME, Italy; marianna.oteri@unime.it (M.O.); luigi.liotta@unime.it (L.L.); gianni.dipasquale@studenti.unime.it (G.D.); vincenzo.chiofalo@unime.it (V.C.); 2 School of Biosciences and Veterinary Medicine, University of Camerino, Via Gentile III da Varano, 62032 Camerino, MC, Italy; daniela.beghelli@unicam.it; 3 Feed Manufacturing Industry, Mangimi Di Pasquale srl, Strada Statale 115 km 384,27 snc, 96012 Avola, SR, Italy; biagiotuve98@gmail.com (B.T.);; 4 Consortium of Research for Meat Chain and Agrifood (CoRFilCarni), Viale Palatucci 13, 98168 Messina, ME, Italy; mariaelena.furfaro@corfilcarni.it; 5 Department for Life Quality Studies, Alma Mater Studiorum—University of Bologna, Rimini Campus, Corso d’Augusto 237, 47921 Rimini, RN, Italy; fabiana.antognoni@unibo.it; 6 Division of Pharmacognosy & Natural Products Chemistry, Department of Pharmacy, National and Kapodistrian University of Athens, Panepistimiopolis of Zographou, 157 71 Athens, Greece; marialianza@pharm.uoa.gr

**Keywords:** beef cattle, olive cake, polyphenols, antioxidant capacity, fatty acid composition, meat quality, circular economy

## Abstract

This study involved 45 finishing Limousin young bulls (initial BW 350 ± 15 kg; aged 250 ± 20 days) reared under commercial conditions and randomly assigned for 250 days to three dietary treatments: a control diet without olive cake (CTR), and diets including partially destoned dried olive cake at 10% (OC10) or 15% (OC15) of dry matter. The effects of the dietary inclusion of partially destoned dried olive cake (DOC) on the physicochemical properties, intramuscular fatty acid composition, nutritional lipid indices, and antioxidant profile of the *Longissimus dorsi* muscle were evaluated. Meat pH was measured at 45 min, 24 h, and 7 days of aging. Instrumental color, proximate composition, fatty acid profile, lipid nutritional indices, total phenolic content (TPC), individual polyphenols, and antioxidant capacity were determined. Data were analyzed by ANOVA using PROC GLIMMIX in SAS, with orthogonal contrasts to evaluate linear and quadratic responses to olive cake inclusion. Treatments did not affect post mortem pH, and color differences observed at 24 h were not present after 7 days of aging. Crude protein content was lower in OC-fed groups, while total lipid content tended to increase; sodium chloride concentration was higher in OC15 meat. Olive cake supplementation reduced hypercholesterolemic saturated fatty acids and increased stearic, oleic, and polyunsaturated fatty acids, including α-linolenic acid, EPA, and DPA, improving PUFA/SFA and UFA/SFA ratios and reducing atherogenic and thrombogenic indices. Despite a higher peroxidability index, OC groups showed greater muscle TPC and antioxidant capacity, the presence of detectable hydroxytyrosol and tyrosol suggests a possible contribution of dietary olive phenolics or their metabolites, although the exact mechanisms underlying their appearance in muscle tissue remain to be fully elucidated. Overall, partially destoned DOC can be included up to 15% of the finishing diet to improve beef nutritional and functional quality within sustainable circular systems.

## Simple Summary

Large quantities of by-products are generated during olive oil production, many of which are still underutilized despite their high nutritional and functional potential. Olive cake is one such by-product, characterized by a valuable content of beneficial fatty acids and natural antioxidant compounds. The aim of this study was to evaluate whether partially destoned dried olive cake can be included in the diets of beef cattle and to assess its effects on meat quality and nutritional value. Limousin bulls in the finishing stage were fed diets containing olive cake at two inclusion levels and compared with animals receiving a conventional diet. The inclusion of olive cake did not negatively affect meat acidity or visual appearance, indicating that overall meat quality was preserved. Conversely, meat from olive cake-fed animals exhibited an improved lipid profile, with higher proportions of fatty acids considered beneficial for human health. Moreover, these animals produced meat with increased concentrations of natural antioxidant compounds derived from olives. Overall, the results demonstrate that olive cake can be effectively used in beef cattle feeding to enhance the nutritional quality of meat while simultaneously promoting more sustainable livestock production through the valorization of agro-industrial by-products.

## 1. Introduction

Cattle production remains a cornerstone of the agri-food sector, and the optimization of nutritional strategies is essential for improving production efficiency, meat quality, and sustainability [1,2,3]. Among beef breeds, Limousin cattle are particularly valued for their adaptability, resilience, and superior carcass traits, including high lean yield and excellent muscular conformation [4]. These characteristics make Limousin bulls an appropriate model for evaluating innovative feeding strategies aimed at enhancing sustainability and animal welfare while maintaining high product quality [5,6].

In this context, the valorization of agro-industrial by-products as functional feed resources has gained increasing attention, as it supports circular economy principles and improves the environmental sustainability of livestock systems [7,8,9]. Among these by-products, partially destoned DOC, a residual material from olive oil extraction, has emerged as a promising dietary ingredient, particularly in Mediterranean regions. This by-product is rich in bioactive compounds, including monounsaturated (MUFA) and polyunsaturated fatty acids (PUFA), phenolic antioxidants, and dietary fiber, which may beneficially influence rumen fermentation, oxidative status, and meat lipid composition [10,11,12,13].

Within this context, regional quality schemes such as “QS Sicilia” promote sustainable livestock production practices by encouraging the use of locally available agro-industrial by-products, such as olive cake, in ruminant diets. The increasing adoption of such quality labels, together with their emphasis on circular economy and resource valorization, highlights the need for robust scientific evidence supporting the nutritional, productive, and qualitative effects of these feed ingredients. This perspective further motivated the present study to deepen the investigation of partially destoned DOC as a functional dietary component in beef cattle production systems.

Several ruminant feeding trials have demonstrated that olive cake can be included in diets without impairing digestive function while positively affecting animal performance and meat quality. For example, Chiofalo V. et al. [14] reported improved average daily gain, carcass weight, intramuscular fat deposition, and meat tenderness in Limousin bulls fed olive cake, together with a more favorable fatty acid profile and reduced cooking losses. Lopreiato et al. [15] further observed modifications in ruminal volatile fatty acid patterns and potential reductions in enteric methane emissions at an inclusion level of 13%, although responses at higher dietary levels require further investigation. In addition, in vitro studies suggest that olive by-products can modulate rumen microbial populations, improving biohydrogenation pathways and fiber degradation while maintaining stable fermentation dynamics [11]. Collectively, these findings indicate that olive cake can be safely included as up to 15–20% of dietary dry matter, with beneficial effects on the lipid composition of both meat and milk [16,17].

The functional potential of olive by-products has also been confirmed in non-ruminant species and dairy livestock. Studies in pigs and dairy animals have reported improvements in performance, metabolic status, and product quality [18,19,20,21]. In beef cattle, Bionda et al. [22] documented improved cholesterol metabolism and stable liver enzyme activity following dietary inclusion of 15% olive cake, supporting its metabolic safety. Similarly, Fazio et al. [23] reported a positive modulation of endocrine responses, further validating olive cake as a functional feed ingredient.

Moreover, performance parameters such as body weight, average daily gain, carcass weight, and dressing percentage were not adversely affected by olive cake inclusion up to 15%, confirming its productive efficacy and metabolic tolerance [22].

Despite these promising results, limited information is available on the dose-dependent effects of partially destoned DOC during the finishing phase of high-value beef breeds. Most studies have focused on the effects of low-to-moderate inclusion levels on general performance outcomes, leaving a knowledge gap regarding post mortem meat quality traits and oxidative stability under practical feeding conditions.

Given the increased susceptibility of PUFA-enriched meat to lipid oxidation, the presence of olive-derived phenolic compounds may play a crucial role in preserving oxidative stability while enhancing the functional value of beef.

Therefore, the present study aimed to investigate the effects of including partially destoned DOC at 10% and 15% of dietary dry matter in finishing diets for Limousin bulls on *post-slaughter* meat quality characteristics.

## 2. Materials and Methods

### 2.1. Ethical Statement

All experimental procedures were approved by the Ethical Committee of the Department of Veterinary Science, University of Messina, Italy (approval code: 041/2020). The study was conducted in compliance with Italian and European regulations on animal welfare, including Directive 2010/63/EU on the protection of animals used for scientific purposes [24], and in accordance with the principles of Good Clinical Practice [25].

### 2.2. Experimental Design, Animals, and Diets

The experimental trial was conducted over a 250-day period (February to September 2021) on a commercial beef cattle farm located in Santa Croce Camerina, in the Hyblean area of Sicily (Italy), characterized by a typical Mediterranean climate. Further details regarding the experimental design and conditions have been previously described by Bionda et al. [22].

A total of 45 Limousin young bulls (aged 250 ± 20 days) were enrolled in the trial. At the beginning of the study, animals had a homogeneous average initial body weight (BW) of 350 ± 15 kg. All bulls were housed on the same farm in three contiguous straw-bedded pens (15 animals per pen), with a space allowance of 3.5 m^2^/head.

Animals were randomly assigned to three dietary treatment groups (15 animals per group): control group (CTR), receiving conventional concentrate with no OC inclusion; low-olive cake group (OC10), with 10% destoned DOC inclusion; and high-olive cake group (OC15), with 15% destoned DOC inclusion. Before starting the trial, animals were fed the CTR concentrate for 15 days as an adaptation phase.

In the CTR diet, a commercial rumen-protected fat product characterized by a high stearic acid (C18:0) content (approximately >80% of total fatty acids) was included in the concentrate to maintain dietary isoenergeticity. The DOC used in this study was partially destoned and enriched with 5% olive leaves.

Animals received their concentrate (equal to 2% of their body weight, on average) twice daily (7.00 a.m. and 3.00 p.m.) and they were allowed ad libitum access to straw and water. The only dietary variable among groups was the level of OC inclusion in the concentrate. Full details of the concentrate formulations have been published previously [22].

Upon arrival, all bulls were vaccinated and treated for internal and external parasites. Veterinary inspections were carried out twice daily and no clinical issues or health disorders were reported during the trial period.

At the end of the feeding period, animals were transported to a certified abattoir located 19.2 km from the farm. Prior to slaughter, bulls were fasted for 12 h in accordance with EU animal welfare regulations. Approximately 30 min post mortem, hot carcass weight was recorded following the procedure described by Cozzi et al. [26]. Carcasses were then stored under controlled refrigeration (0–4 °C, 62–87% relative humidity) and aged for seven days prior to sampling.

### 2.3. Chemical Characterization of Feeds: Proximate Composition, Fatty Acids, Phenolics, and Antioxidant Capacity

Feed samples were collected at two key stages of the experimental period, at the start of the fattening phase (day 15) and at the start of the finishing phase (150 days after the beginning of fattening), from all batches of concentrates and wheat straw provided to each treatment group (CTR, OC10, and OC15). For each time point and feed component, composite samples (*n* = 3) were prepared by pooling sub-samples representative of the entire feeding period. Additionally, a representative pooled sample of the DOC, enriched with 5% olive leaves, was obtained by combining material collected during each sampling phase.

All samples (concentrates, straw, and DOC) were analyzed for dry matter (DM), crude protein (CP), ether extract (EE), and ash content following standardized procedures established by the Association of Official Analytical Chemists (AOAC) [27]: DM (method 930.15, 1999), CP (method 2001.11, 2005), EE (method 920.39, 1920), and ash (method 942.05, 1942).

Fiber fractions, including neutral detergent fiber (NDF), acid detergent fiber (ADF), and acid detergent lignin (ADL), were determined in straw samples according to the method described by Goering and Van Soest [28].

For fatty acid (FA) profiling, lipids were extracted from concentrates and DOC, followed by direct transesterification to fatty acid methyl esters (FAMEs), as described by Christie [29]. Analyses were conducted using a gas chromatograph equipped with a flame ionization detector (GC-FID, TRACE 1310, Thermo Fisher Scientific, Milan, Italy) and an Omegawax 250 capillary column (Supelco, Bellefonte, PA, USA), following the procedure of Oteri et al. [30]. Fatty acid identification and quantification were performed using Chromeleon™ Data System Software (version 7.2.9, Thermo Fisher Scientific). Peaks were identified by comparison with a certified 37-FAME standard mix (Supelco). Results are expressed as grams per 100 g of total identified FAMEs.

To evaluate the bioactive properties of the experimental diets, methanolic extracts were prepared from DOC and supplemented concentrates’ samples (OC10 and OC15, 5 g each) using a Soxhlet apparatus for 5 h at 65 °C with 200 mL of pure methanol. The obtained solutions were then evaporated with a Rotavapor Buchi B-490 (Büchi Labortechnik AG, Flawil, Switzerland) for the characterization of the final extracts.

Antioxidant activities in terms of radical scavenging activity were evaluated using the 1,1-diphenyl-2-picrylhydrazyl (DPPH) and the 2,2′-azino-bis(3-ethylbenzothiazoline-6-sulphonate) diammonium salt (ABTS) methods, and results were expressed as Trolox equivalent (mg TE/5 g sample) [31,32,33].

The total phenolic content (TPC) determination in the same extracts was carried out using the Folin–Ciocalteu method, and values were expressed as mg of gallic acid equivalents (GAE) per 5 g of sample weight (mg GAE/5 g sample) [34].

Characterization of individual polyphenols (hydroxytyrosol, tyrosol, oleuropein, apigenin, luteolin, myricetin, pinoresinol, and ligstroside) in methanolic extracts of DOC and DOC-enriched concentrates was carried out following the procedures reported by Alimenti et al. [33] using a Jasco HPLC-DAD system, consisting of a PU-4180 pump, an MD-4015 PDA detector, and an AS-4050 autosampler (Jasco Corporation, Hachioji, Tokyo, Japan). An Agilent Zorbax Eclipse Plus C18 reverse-phase column (Agilent, Santa Clara, CA, USA; 4.6 × 100 mm I.D, 3.5 μm) was used as the stationary phase. However, DOC and complete diets (OC10 and OC15) differ in matrix composition and it should be noted that differences in feed matrix complexity may influence the extraction efficiency and the analytical recovery of individual phenolic classes, thus explaining differences between the two types of samples [35]. Data are expressed as means of mg compound/Kg extract ± SD.

The proximate composition of the concentrates and straw is presented in Table 1. The chemical composition and fatty acid profile of the destoned DOC included in the experimental diets are shown in Table 2, the fatty acid profile of the concentrates is detailed in Table 3, while the antioxidant scavenging properties, TPC, and phenolic characterization of the methanolic extracts are represented in Table 4.

### 2.4. In Vivo and Post Mortem Parameters

In vivo performance parameters, including body weight (BW) and average daily gain (ADG), were previously reported in detail by Bionda et al. [22]. Briefly, differences between diet groups were observed: body weight was higher in the CTR group, although average daily gain and carcass weight were similar across all diets. Dressing percentage was higher in OC15 bulls, with OC10 bulls showing intermediate values. These observations indicate that the finishing diet had a notable impact on the carcass traits of bulls.

At the end of the feeding trial, all bulls were slaughtered humanely in a licensed abattoir, in full compliance with European Union legislation on the protection of animals at the time of harvesting (Regulation (EC) No 1099/2009) [36].

Approximately 30 min post mortem, hot carcass weight (HCW) was recorded following the removal of the hide, head, limbs, and internal organs. Dressing percentage was calculated as the ratio between HCW and final live BW. Carcass traits, including HCW and dressing percentage, were previously reported by Bionda et al. [22] and are therefore not presented in this manuscript.

### 2.5. Meat Sample Collection and Analysis of Physical, Chemical, and Nutritional Traits

To evaluate meat quality characteristics, individual samples of the *Longissimus dorsi* muscle were collected from each animal 24 h post mortem. Specifically, muscle sections were excised between the 7th and 8th thoracic vertebrae, yielding a total of 45 samples (*n* = 15 per treatment group: CTR, OC10, and OC15). Each sample (approximately 300–400 g) was vacuum-sealed in food-grade plastic bags and immediately transported under refrigerated conditions (4 ± 1 °C) to the analytical facilities of the Meat and Agribusiness Chain Research Consortium (Co.R.Fil.Carni) for laboratory analyses.

Muscle pH and temperature were measured at 45 min (pH_1_) and 24 h (pH_2_) post mortem for the *Longissimus thoracis* muscle between the 12th and 13th ribs. Measurements were conducted using calibrated portable pH meters (WTW 330/SET 1, Weilheim, Germany; Hanna Instruments, Woonsocket, RI, USA) equipped with penetrating glass electrodes, including the Hamilton Double-Pore model, specifically designed for meat analysis. The probes were inserted approximately 5 cm into the geometric center of the muscle to obtain representative internal values. Temperature was recorded at the same depth to monitor the rate of post mortem cooling. These parameters were used to assess glycolytic activity and classify meat quality in relation to pH-dependent alterations. Temperature data were not reported in this study, as they were used solely to support the interpretation of pH evolution.

In addition to early post mortem measurements, ultimate pH was also assessed after 7 days of refrigerated aging on the *Longissimus dorsi* samples (pH*(7 days)). This value was used as an indicator of final muscle acidity and to further evaluate the progression of post mortem biochemical changes during aging.

The chemical–nutritional composition of ground *Longissimus dorsi* samples was determined in triplicate (*n* = 45) using AOAC International official methods [27]. The following parameters were analyzed: moisture content, AOAC 950.46 (2010); ash content, AOAC 920.153 (1920) and 923.03 (1923); crude protein, AOAC 981.10 (1983) via Kjeldahl nitrogen determination; and total lipid content, AOAC 991.36 (1996) via Soxhlet extraction. All values were expressed on a fresh weight basis (g/100 g of meat).

Salt content (NaCl) was determined using Near-Infrared Spectroscopy (NIRS) in transmittance mode with a calibrated FoodScan Meat Analyzer (FOSS Analytical, Padova, Italy), following the manufacturer’s protocol. This non-destructive technique enables the rapid and accurate quantification of sodium chloride based on molecular absorption in the NIR spectrum, with results expressed as % NaCl (*w*/*w*).

This set of analyses provided a comprehensive overview of the basic nutritional profile of the meat, essential for both meat quality assessment and nutritional labeling, and for evaluating the impact of dietary olive cake inclusion on beef composition.

### 2.6. Meat Color Analyses

The colorimetric evaluation of meat was performed on *Longissimus dorsi* muscle samples to investigate the effects of dietary treatments on surface color development over time. All measurements were carried out using the CIE Lab color space system, which defines three primary attributes of meat color: L* (lightness), a* (redness), and b* (yellowness). In addition, two derived indices were calculated to provide a more comprehensive assessment of meat color characteristics: chroma (C*), which reflects color saturation, and hue angle (H°), which indicates the color tone or red-to-yellow balance. These parameters are particularly useful for detecting color shifts influenced by oxidative status and the presence of dietary antioxidants such as phenolic compounds.

Color measurements were conducted at two critical post mortem stages:

- At 24 h after slaughter, corresponding to the early retail phase and denoted as L* (24 h), a* (24 h), b* (24 h), C* (24 h), and H° (24 h);
- After 7 days of refrigerated aging, to evaluate the impact of maturation on meat color stability, indicated as L* (7 days), a* (7 days), b* (7 days), C* (7 days), and H° (7 days).

At both time points, 20 mm-thick slices of the *Longissimus dorsi* were freshly cut and exposed to ambient air at 4 °C for 30 min (blooming phase) to allow oxygenation of myoglobin, following the protocol described by Avilés et al. [37]. Surface color was measured using a portable spectrocolorimeter (Konica Minolta CM-600d, Chiyoda, Japan) at five different points per sample, carefully avoiding visible fat or connective tissue. Prior to each session, the instrument was calibrated using a standard white tile provided by the manufacturer.

The following formulas were applied to calculate derived indices, according to ASPA guidelines [38]:

Chroma (C*), indicating color saturation or intensity:

C* = ((a*2 + b*2)1/2)

Hue angle (H°), representing color tone (i.e., the red–yellow balance):

H° = (tan − 1 (b*/a*))

These colorimetric assessments, conducted both at 24 h and after 7 days of aging, allowed for a dynamic evaluation of post mortem changes in meat appearance and their potential modulation by dietary inclusion of olive cake.

### 2.7. Fatty Acid Composition and Nutritional Quality Parameters of Longissimus dorsi

Total lipids were extracted from homogenized *Longissimus dorsi* samples using the Soxhlet method (AOAC 991.36) [27]. Fatty acid methyl esters (FAMEs) were subsequently prepared through direct transesterification using methanolic acid under controlled heating conditions, as described by Christie [29], to ensure complete esterification of all lipid fractions.

Fatty acid profiles were analyzed by gas chromatography with flame ionization detection (GC-FID) (TRACE 1310, Thermo Fisher Scientific, Milan, Italy). Separation was achieved on a high-polarity capillary column (Omegawax 250, 30 m × 0.25 mm i.d., 0.25 µm film thickness; Supelco, Bellefonte, PA, USA), using analytical conditions adapted from Oteri et al. [39] for high-resolution separation of saturated (SFA), monounsaturated (MUFA), and polyunsaturated fatty acids (PUFA).

Chromatographic data were processed using Chromeleon Data System Software (version 7.2.9, Thermo Fisher Scientific). Fatty acid identification was achieved by comparing retention times with those of a certified 37-component FAME standard mixture (Supelco 47885-U, Bellefonte, PA, USA). Results are expressed as g/100 g of total identified FAMEs.

Fatty acid classes were grouped as follows: total saturated fatty acids (SFA); total monounsaturated fatty acids (MUFA); total polyunsaturated fatty acids (PUFA), including both n-6 and n-3 series and PUFA n-3/n-6 ratio.

To assess the health-related properties of meat lipids, the following nutritional indices were calculated:

Atherogenic Index (AI), which reflects the potential for promoting atheroma formation [40]:

AI = [C12:0 + (4 × C14:0) + C16:0]/(Ʃn6-PUFA + Ʃn3-PUFA + ƩMUFA),

Thrombogenic Index (TI), which estimates the tendency to form blood clots [40]:

TI = (C14:0 + C16:0 + C18:0)/[(0.5 × ƩMUFA) + (0.5 × Ʃn6-PUFA) + (3 × Ʃn3-PUFA) + (Ʃn3-PUFA/Ʃn6-PUFA)],

Hypocholesterolemic/Hypercholesterolemic ratio (h/H), which indicates the balance of cholesterol-lowering vs. cholesterol-raising fatty acids [41]:

h/H = (C18:1n9 + C18:2n6 + C20:4n6 + C18:3n3 + C20:5n3 + C22:5n3 + C22:6n3)/ (C14:0 + C16:0)

These indices are essential for evaluating the nutritional quality of meat lipids, especially in light of growing consumer demand for healthier animal products with improved fatty acid profiles and functional properties.

Additionally, the Peroxidation Index (PI) was calculated to estimate the oxidative susceptibility of the fatty acid profile [42]:

PI = (% dienoic × 1) + (% trienoic × 2) + (% tetraenoic × 3) + (% pentaenoic × 4) + (% hexaenoic × 5)

This index provides insight into the oxidative stability of meat lipids, with direct implications for both shelf-life and nutritional integrity.

### 2.8. Determination of Total Phenolic Content, Polyphenol Composition, and Antioxidant Capacity in Meat

Meat samples (*Longissimus dorsi*) were collected at the abattoir, kept protected from light, and transported frozen to the laboratory. Samples were stored at −20 °C and maintained frozen until analysis, which was performed within one week, in order to preserve phenolic compounds and limit their degradation. Aliquots of meat were reduced to fragments of a few millimeters on ice and subsequently frozen in liquid nitrogen. For each experimental group, a pool of equal amounts of meat (5 g) obtained from 3 animals was combined to generate a representative composite sample per treatment. Meat was ground in methanol (20 mL) using a Turrax T25 grinding machine (IKA-Werke GmbH & Co. KG, Staufen, Germany). Successively, the homogenates were wrapped in laboratory filter paper (Qual Pia D.150 mm) and extracted by Soxhlet for 5 h at 65 °C, using a final methanol volume of 250 mL. The extracts were then evaporated with a Rotavapor Buchi B-490 instrument to remove the remaining solvent and analyzed as previously described to evaluate the radical scavenging activities, total phenol content, and characterization of polyphenols. This approach was adopted to obtain an overall treatment-level phenolic profile, even if it does not allow the estimation of inter-individual biological variability within groups. Each pooled extract was analyzed in technical triplicate.

### 2.9. Statistical Analysis

Statistical analysis of physiochemical traits, fatty acid profile, phenolic composition, total phenolic content, and antioxidant activity was performed in SAS (ver. 9.4, SAS Institute Inc., Cary, NC, USA). Sample size was based on the availability of animals in the herd and supported by a power analysis (POWER procedure of SAS). From previous studies with similar experimental designs, a sample size of 15 subjects/group would be sufficient. Before analysis, the normality of distributions was checked by calculating the kurtosis and skewness of the data (UNIVARIATE procedure of SAS). Data were subjected to ANOVA and analyzed using the GLIMMIX procedure. Data were analyzed using a model that included levels of inclusion [CTR (0%), OC10 (10%), or OC15 (15%)] as fixed effects and pen as a random effect, with animals nested within pen. The individual animal was considered the experimental unit for the analyzed response variables. The normality of the residuals was tested via PROC UNIVARIATE in SAS. All observations were normally distributed. The Kenward–Roger statement was used for computing the denominator degrees of freedom. Orthogonal contrasts were used to determine the linear or quadratic effect of inclusion level [CTR (0%), OC10 (10%), or OC15 (15%)] for fatty acid profile data. Tukey’s test was used for multiple comparisons, and linear and quadratic trends were further assessed. Differences were regarded as statistically significant when *p* ≤ 0.05.

## 3. Results

### 3.1. Physicochemical Traits and Proximate Composition of Longissimus dorsi

Physicochemical parameters of meat, including pH measured at 45 min (pH_1_), 24 h (pH_2_), and after 7 days of aging (pH*), along with chemical composition, were assessed in triplicate for each sample. Least Squares Means (LSM ± SEM) are reported in Table 5.

No significant differences were found for pH_1_ or pH_2_ across treatment groups, indicating a comparable rate of post mortem glycolysis and consistent muscle acidification dynamics. Similarly, the ultimate pH measured after 7 days of refrigerated aging (pH*) did not differ significantly among groups (*p* = 0.3306), suggesting that dietary olive cake inclusion did not influence final muscle acidity.

Among the colorimetric indices measured at 24 h, L* and b* showed significant differences across groups (L* *p* = 0.0085; b* *p* = 0.0107), with OC15 showing lower L* and b* values. In contrast, chroma (C*) and hue angle (H°) did not differ significantly (C* *p* = 0.1003; H° *p* = 0.1201), although a trend towards increased H° was noted in OC10, possibly reflecting subtle shifts in color tone due to dietary components.

After 7 days of aging, neither C* nor H° showed significant treatment effects (C*, *p* = 0.2000; H°, *p* = 0.1210), suggesting that post mortem maturation did not exacerbate or mitigate any initial differences related to the dietary treatments.

As for the chemical composition, crude protein (CP) was significantly reduced in both OC10 (24.42%) and OC15 (24.84%) groups compared to the CTR group (25.65%; *p* = 0.0092), reflecting a potential dilution effect or altered muscle deposition linked to olive cake inclusion. Total lipid content (TL) tended to increase in the OC10 group (2.27%), although the difference was not statistically significant (*p* = 0.1557).

No significant differences were observed in ash content among treatments. However, sodium chloride (NaCl) concentration was significantly higher in the OC15 group (1.15%) compared to CTR (1.03%) and OC10 (1.01%) (*p* = 0.0114), possibly due to compositional differences in feed ingredients or alterations in muscle ion retention.

Taken together, these findings suggest that the dietary inclusion of dried olive cake, particularly at higher levels, may influence certain physicochemical traits of *Longissimus dorsi* muscle, notably crude protein content and salt concentration, without substantially affecting post mortem pH or core color stability indicators such as chroma and hue.

### 3.2. Fatty Acids, Acidic Classes, and Nutritional Indices of Longissimus dorsi

The effects of dietary inclusion of partially destoned DOC on the intramuscular fatty acid composition of *Longissimus dorsi* muscle in Limousin bulls are presented in Table 6. The table reports Least Squares Means (LSM) for individual fatty acids, grouped fatty acid classes, as well as nutritional indices. These variables provide a comprehensive evaluation of the lipid profile and health-related properties of the meat in response to dietary treatments.

The inclusion of OC at both 10% and 15% significantly affected the fatty acid composition. A marked reduction was observed in several SFA, particularly myristic acid (C14:0; *p* = 0.0119) and palmitic acid (C16:0; *p* < 0.0001), both associated with hypercholesterolemic effects. In contrast, stearic acid (C18:0) increased significantly (*p* < 0.0001), potentially reflecting altered ruminal biohydrogenation processes. As a result, total SFA content declined significantly with OC supplementation (*p* = 0.0005).

Among MUFA, although some components such as myristoleic acid (C14:1) and palmitoleic acid (C16:1) decreased, oleic acid (C18:1n9) significantly increased (*p* = 0.002). This led to a significant rise in overall MUFA levels (*p* = 0.0133), especially in the OC15 group, contributing to an improved lipid profile.

PUFA levels also rose significantly with increasing OC inclusion (*p* = 0.0003). Notable increases were detected for alpha-linolenic acid (C18:3n3; *p* < 0.0001), eicosapentaenoic acid (C20:5n3; *p* = 0.0179), and docosapentaenoic acid (C22:5n3; *p* = 0.0413). Enhancements were also observed in the n-6 series, including linoleic acid (C18:2n6; *p* = 0.0042) and arachidonic acid (C20:4n6; *p* = 0.0062). The PUFA/SFA ratio (*p* < 0.0001) and UFA/SFA ratio (*p* = 0.0007) improved significantly, indicating a shift toward a more unsaturated fatty acid profile.

The n6/n3 ratio decreased significantly from 4.40 in CTR to 3.42 in OC10 and 3.29 in OC15 (*p* = 0.0016), showing a dose-dependent quadratic response (*p* = 0.0363). This reduction reflects a stronger relative increase in n-3 PUFA compared with n-6 PUFA, thus improving the balance between these two fatty acid classes and enhancing the nutritional quality of the meat.

In terms of nutritional indices, both the atherogenic index (AI) and thrombogenic index (TI) were significantly reduced by OC supplementation (*p* < 0.0001), while the hypocholesterolemic/hypercholesterolemic ratio (h/H) increased significantly (*p* < 0.0001). The peroxidation index (PI) also increased (*p* = 0.0017), likely reflecting the higher PUFA content and greater susceptibility to oxidation.

In summary, dietary supplementation with partially destoned DOC at 10% and 15% significantly improved the lipid quality of beef by reducing atherogenic and thrombogenic saturated fatty acids, while enhancing the proportion of beneficial unsaturated fatty acids and associated health indices. These findings support the potential of olive by-products as functional feed ingredients in beef production systems aimed at enhancing meat nutritional value.

### 3.3. Total Phenolic Content, Polyphenols, and Antioxidant Activity of Longissimus dorsi

The effects of dietary inclusion of partially destoned DOC on the radical scavenging activities and polyphenol content in *Longissimus dorsi* muscle extracts of Limousin bulls are presented in Table 7.

Among the eight phenolic compounds investigated, hydroxytyrosol and tyrosol were the only ones detectable. However, dietary supplementation with DOC at 10% and 15% proportionally and significantly improved their contents in beef, enhancing the proportion of health-promoting bioactive compounds [43,44,45,46].

## 4. Discussion

The present study provides novel insights into the dose-dependent effects of partially destoned dried olive cake (DOC) inclusion on beef quality traits in Limousin bulls, reinforcing its potential as a functional feed ingredient capable of enhancing the nutritional profile of beef while preserving animal metabolic health, as previously reported by Bionda et al. [22].

To our knowledge, few studies have explored the dose-dependent inclusion of partially destoned DOC within high-value beef production systems, and the present work contributes new evidence on its effects on meat quality traits, fatty acid composition, and antioxidant characteristics.

Regarding the physicochemical traits and proximate composition of the *Longissimus dorsi* muscle, OC inclusion up to 15% did not significantly alter fundamental meat quality parameters such as post mortem pH, confirming the metabolic neutrality of OC on muscle glycolysis and lactic acid accumulation [42,47]. However, minor but significant reductions in crude protein content and increased sodium chloride levels in the highest OC group were observed. The decrease in crude protein may be related to a dilution effect due to the higher fiber content and partial replacement of conventional concentrate with olive cake, which can increase the relative contribution of non-protein components (e.g., fiber and lipids) in muscle tissue. Moreover, OC inclusion could modify dietary energy density and ruminal fermentation, potentially affecting nutrient partitioning and muscle protein deposition. The tendency for higher intramuscular fat in OC-fed groups could also contribute to a lower protein percentage, as increased lipid deposition reduces the relative proportion of protein. These interpretations remain hypotheses and require further mechanistic investigation to confirm the underlying physiological mechanisms.

The increased sodium chloride content in OC15 meat could be related to the mineral composition of the olive cake and its effects on electrolyte balance [42,47,48,49]. However, dietary sodium levels were not significantly different among treatments and mineral intake was not directly measured; therefore, this result should be considered an unexpected finding that warrants further investigation to elucidate the underlying mechanisms.

Significant differences in L* and b* at 24 h were not maintained after 7 days of aging, suggesting limited biological and commercial relevance. These early differences may reflect a transient antioxidant effect on myoglobin oxidation, potentially delaying color changes under retail conditions rather than providing long-term stability.

The most prominent effects of OC supplementation emerged in the intramuscular fatty acid composition. Dietary OC significantly reduced hypercholesterolemic saturated fatty acids, particularly myristic (C14:0) and palmitic acid (C16:0), while increasing beneficial monounsaturated and polyunsaturated fatty acids, including oleic acid (C18:1n9), α-linolenic acid (C18:3n3), eicosapentaenoic acid (EPA), and docosa-pentaenoic acid (DPA). These alterations are presumably driven by the bioactive phenolic compounds in OC, which may modulate ruminal biohydrogenation pathways and enhance endogenous desaturation activity, as supported by the concomitant rise in stearic acid (C18:0), a key hydrogenation intermediate [47,50,51,52].

These changes also resulted in a progressive decrease in the n-6/n-3 ratio, showing a dose-dependent response to increasing OC inclusion. The reduction was more marked at the initial supplementation level, with a further, smaller improvement at the higher inclusion rate, reflecting a stronger relative increase in n-3 PUFA compared with n-6 PUFA. A lower n-6/n-3 ratio is widely considered beneficial for human health, as it is associated with reduced inflammatory potential and improved cardiovascular outcomes. Therefore, the dose-dependent modulation of the n-6/n-3 balance further supports the use of OC as a functional feed ingredient to enhance the nutritional value of beef.

These changes resulted in significant improvements in health-related lipid indices, including the atherogenic (AI) and thrombogenic (TI) indices, hypocholesterolemic/hypercholesterolemic ratio (h/H), and both UFA/SFA and PUFA/SFA ratios. Collectively, these shifts reflect a more cardio-protective fatty acid profile [53,54,55,56], in alignment with current nutritional recommendations for red meat fat quality [57,58]. Although OC inclusion increased the peroxidation index (PI), likely due to higher PUFA content, the polyphenolic compounds present in OC may mitigate lipid oxidation, contributing to improved oxidative stability and extended shelf life [42,59]. In addition to lipid protection, this antioxidant effect could positively influence myoglobin stability and thus meat color, a key quality parameter that strongly impacts consumer perception at retail. Indeed, early post mortem color characteristics have been proposed as predictors of oxidative color stability during aging [60]. This highlights the potential of OC as a natural alternative to synthetic preservatives for maintaining both lipid and pigment stability in beef.

Consistently, meat from OC-supplemented animals showed significantly higher total antioxidant activity and total phenolic content compared with the control [61]. While no differences were observed between the 10% and 15% inclusion levels for global antioxidant indices [62], hydroxytyrosol and tyrosol concentrations increased in a dose-dependent manner, indicating a selective enrichment of olive-derived phenolic compounds in muscle tissue [63].

The phenolic compounds, including oleuropein, tyrosol, and hydroxytyrosol, which are main components of olive cake, can be released or transformed during feed processing and ruminal metabolism. Dietary inclusion of olive by-products in ruminant feeding has been shown to influence rumen function, animal metabolism, and the profile of bioactive compounds in derived products such as milk, suggesting that phenolic compounds and/or their metabolites may be absorbed and distributed systemically in the animal [17].

Furthermore, bioavailability studies indicate that hydroxytyrosol and related polyphenols undergo digestion, absorption and metabolic transformation in animals [64]. While direct analysis of hydroxytyrosol and tyrosol in bovine muscle following dietary supplementation remains limited, the general metabolic pathways and presence of these compounds in animal products provide a mechanistic basis for their detection in beef. However, as lipid oxidation was not directly assessed in the present study, any inference regarding oxidative stability remains indirect and warrants further investigation.

In addition, a methodological limitation of the present study is that phenolic analyses were performed on pooled samples per dietary group. While this strategy provides a representative treatment-level profile, it precludes the assessment of inter-individual biological variability and limits the robustness of statistical inference among treatments. Therefore, differences in phenolic concentrations among groups should be interpreted with caution and confirmed in future studies including independent biological replicates.

Furthermore, the enhancement of lipid quality, particularly the increased oleic acid and PUFA levels, could also improve sensory attributes such as flavor and juiciness, which are positively associated with consumer preference [65,66].

Overall, these findings support the feasibility of incorporating olive industry by-products into ruminant feeding programs as part of a circular economy strategy. The dose-dependent improvements in beef lipid quality and functional indices demonstrate the potential for sustainable livestock production systems that align nutritional enhancement with environmental valorization [18,19].

Moreover, given its abundance and generally low cost, OC may represent a scalable feed resource, supporting economically viable sustainability transitions in beef production.

It should be acknowledged that this study was conducted using a single beef breed within a single farm, which may limit the generalizability of the results; however, the Limousin breed represents a widely used and highly relevant model for beef production, supporting the applicability of the findings to comparable production systems.

Future research should investigate long-term oxidative stability, perform detailed sensory and consumer acceptance assessments, and explore the applicability of these results across different cattle breeds, production systems, and feeding environments. Additionally, molecular studies into ruminal biohydrogenation and muscle lipid metabolism are warranted to further optimize the use of OC in functional beef production.

## 5. Conclusions

This study demonstrates that the inclusion of partially destoned DOC up to 15% in the diets of Limousin bulls can improve the nutritional quality of beef without compromising key meat quality traits. OC supplementation positively modulated the intramuscular fatty acid profile by reducing saturated fatty acids and increasing beneficial mono- and polyunsaturated fatty acids, resulting in a more favorable lipid profile for human health. Although minor changes in crude protein content and color parameters were observed, no adverse effects on *post-mortem* biochemical processes were detected. Overall, these findings support the potential use of olive cake as a sustainable functional feed ingredient to enhance beef nutritional value within circular economy frameworks. Future research should further investigate long-term oxidative stability, sensory acceptance, and economic feasibility to confirm the practical applicability of this feeding strategy.

## Figures and Tables

**Table 1:** Proximate composition of concentrates and straw (% dry matter basis).

Item	Concentrates						Straw
	Group (Fattening)			Group (Finishing)			
	CTR	OC10	OC15	CTR	OC10	OC15	
DM	87.44	89.93	90.10	88.09	89.58	89.57	89.21
CP	22.20	18.75	17.77	18.20	18.45	17.99	6.65
EE	3.98	4.82	5.36	5.00	5.88	5.81	1.06
CF	9.31	7.33	7.34	7.34	6.67	6.61	32.85
Ash	8.39	6.13	5.97	7.48	6.71	7.22	4.75
NDF							54.25
ADF							34.16
ADL							4.27

Concentrate: CTR—no inclusion of olive cake; OC10—inclusion of 10% of olive cake; OC15—inclusion of 15% of olive cake; DM = dry matter; CP = crude protein; EE = ether extract; CF = crude fiber; NDF = neutral detergent fiber; ADF = acid detergent fiber; ADL = acid detergent lignin.

**Table 2:** Chemical composition and fatty acid profile of destoned dried olive cake (DOC) used in the trial (% on dry matter basis, % DM).

Item	Destoned DOC
Moisture	6.00
CP	10.02
EE	15.15
CF	27.18
Ash	3.79
pH	4.09
Acidity	1.62
Peroxide number	8.80
*Fatty acid composition*	
C14:0	0.09
C16:0	17.07
C16:1	0.47
C17:0	0.29
C17:1	0.28
C18:0	3.59
C18:1n9	66.42
C18:2n6	9.90
C18:3n3	0.59
C20:0	0.55
C20:1	0.76
SFA	21.58
MUFA	67.93
PUFA	10.49
UFA/SFA	3.63

CP = crude protein; EE = ether extract; CF = crude fiber; C14:0 = myristic acid; C16:0 = palmitic acid; C16:1 = palmitoleic acid; C17:0 = eptadecanoic acid; C17:1 = eptadecenoic acid; C18:0 = stearic acid; C18:1n9 = oleic acid; C18:2n6 = linoleic acid; C18:3n3 = alfa-linolenic acid; C20:0 = arachidic acid; C20:1 = eicosenoic acid; SFA = saturated fatty acids; MUFA = monounsaturated fatty acids; PUFA = polyunsaturated fatty acids; UFA/SFA = unsaturated fatty acids/saturated fatty acids ratio.

**Table 3:** Fatty acids of nutritional interest, fatty acid classes (g/100 g FAME), and UFA/SFA ratio in the concentrates.

	Concentrates—Group (Finishing)		
Item	CTR	OC10	OC15
C14:0	0.15	0.39	0.19
C16:0	32.09	26.78	20.67
C16:1	0.19	0.40	0.36
C17:0	0.09	0.10	0.00
C17:1	0.10	0.09	0.00
C18:0	26.36	9.11	6.02
C18:1n9	21.23	35.15	38.39
C18:2n6	17.85	24.21	29.69
C18:3n3	0.88	1.27	2.24
C20:0	0.95	2.09	2.08
C20:1	0.11	0.42	0.35
SFA	59.64	38.46	28.96
MUFA	21.63	36.05	39.11
PUFA	18.73	25.48	31.93
UFA/SFA	0.68	1.60	2.45

Concentrate: CTR—no inclusion of olive cake; OC10—inclusion of 10% of olive cake; OC15—inclusion of 15% of olive cake. C14:0 = myristic acid; C16:0 = palmitic acid; C16:1 = palmitoleic acid; C17:0 = eptadecanoic acid; C17:1 = eptadecenoic acid; C18:0 = stearic acid; C18:1n9 = oleic acid; C18:2n6 = linoleic acid; C18:3n3 = alfa-linolenic acid; C20:0 = arachidic acid; C20:1 = eicosenoic acid; SFA = saturated fatty acids; MUFA = monounsaturated fatty acids; PUFA = polyunsaturated fatty acids; UFA/SFA = unsaturated fatty acids/saturated fatty acids ratio.

**Table 4:** Scavenging properties (DPPH and ABTS expressed as mg TE/Kg extract), TPC (mg Ac. Gal Eq/g extract) and phenolic characterization (expressed as averages of mg compound/Kg extract ± SD) of the methanolic extracts obtained from DOC and OC10 and OC15 diets. Non-linear differences between the ingredient and the complete diets may arise from matrix effects influencing extraction efficiency and analytical response of individual phenolic compounds. Values are reported as mean ± standard deviation.

Item	DOC	OC10	OC15
TPC	163.9 ± 30.3	80.9 ± 11.4	99.1 ± 12.7
DPPH	48.3 ± 1.88	28.3 ± 1.73	23.4 ± 0.26
ABTS	71.1 ± 1.58	29.1 ± 3.88	36.2 ±1.86
Hydroxytyrosol	188 ± 2.3	171.2 ± 11.1	361.6 ± 3.6
Tyrosol	506.5 ± 5.1	91.8 ± 4.9	143.3 ± 4.5
Oleuropein	332.2 ± 6.4	68.5 ± 6.1	356.9 ± 3.1
Ligstroside	3123.1 ± 4.1	2288.9 ± 3.4	3293.8 ± 3.1
Pinoresinol	133.8± 11.6	104.6 ± 5.9	112.1 ± 11.1
Myricetin	372.0 ± 1.6	363.1 ± 0.3	775.1 ± 5.5
Luteolin	2594.6 ± 12.4	775.1 ± 5.5	528.5 ± 4.9
Apigenin	819.7 ± 5.1	481.4 ± 2.9	493.7 ± 1.5

TPC = total phenolic content; DPPH = 1,1-diphenyl-2-picrylhydrazyl scavenging activity; ABTS = 2-2′-azino-bis (3-ethylbenzo-thiazoline-6-sulphonate) diammonium salt scavenging activity.

**Table 5:** Physicochemical traits and proximate composition (LSM) of beef from bulls fed olive cake-supplemented diets. CTR: control group, receiving conventional concentrate with no olive cake (OC) inclusion; OC10: low-olive cake group, with 10% destoned dried olive cake (DOC) inclusion; OC15: high-olive cake group, with 15% DOC inclusion.

	Group				*p*-Value		
Item	CTR	OC10	OC15	SEM	Group	Lin	Quad
pH_1_	6.56	6.57	6.41	0.19	0.59	0.51	0.61
pH_2_	5.72	5.83	5.82	0.06	0.27	0.13	0.31
pH* (7 days)	5.57	5.55	5.53	0.02	0.33	0.16	0.94
L* (24 h)	37.32 ^a^	38.04 ^a^	34.88 ^b^	0.94	0.01	0.05	0.05
a* (24 h)	9.22	8.57	8.30	0.97	0.71	0.43	0.83
b* (24 h)	13.22 ^a^	14.10 ^a^	11.69 ^b^	0.73	0.01	0.09	0.03
C* (24 h)	16.08	16.51	14.35	1.04	0.10	0.25	0.12
H° (24 h)	55.39	58.99	54.83	1.30	0.12	0.07	0.05
L* (7 days)	38.93	37.87	37.94	1.49	0.82	0.57	0.69
a* (7 days)	9.67	11.60	8.86	1.54	0.19	0.66	0.13
b* (7 days)	15.96	16.73	15.39	1.41	0.58	0.73	0.43
C* (7 days)	18.62	20.38	17.78	2.61	0.20	0.27	0.20
H° (7 days)	59.16	55.23	60.53	1.77	0.12	0.09	0.06
Water Content	72.90	73.35	72.98	0.44	0.60	0.87	0.33
CP	25.65 ^a^	24.42 ^b^	24.84 ^b^	0.25	0.01	0.02	0.01
TL	1.53	2.27	1.70	0.35	0.16	0.67	0.06
Ash	1.13	1.14	1.14	0.05	0.95	0.76	0.85
Salt	1.03 ^b^	1.01 ^b^	1.15 ^a^	0.04	0.01	0.04	0.07

pH_1_ = measured at 45 min *post-sampling*; pH_2_ = measured at 24 h *post-sampling*; L (24 h), a (24 h), and b (24 h) = lightness, redness, and yellowness, respectively, measured at 24 h *post-sampling*; L*, a*, and b* = lightness, redness, and yellowness, respectively, measured after 7 days of aging; C* = chroma calculated after 7 days of aging; H° = hue angle calculated after 7 days of aging; CP = crude protein; TL = total lipids; pH* = pH measured after 7 days of aging. ^a,b^ Different superscript letters indicate significant differences between groups at *p* < 0.05.

**Table 6:** Least Squares Means (LSM) of individual fatty acids, fatty acid classes, and nutritional indices in intramuscular fat from *Longissimus dorsi* muscle of Limousin bulls fed olive cake-supplemented diets. CTR: control group, receiving conventional concentrate with no olive cake (OC) inclusion; OC10: low-olive cake group, with 10% destoned dried olive cake (DOC) inclusion; OC15: high-olive cake group, with 15% DOC inclusion.

	Group				*p*-Value		
Item	CTR	OC10	OC15	SEM	Group	Lin	Quad
C14:0	2.90 ^a^	2.60 ^a^	2.32 ^b^	0.14	0.01	0.01	0.91
C14:1	0.59 ^a^	0.33 ^b^	0.26 ^c^	0.03	<0.0001	<0.0001	<0.001
C15:0	0.50 ^a^	0.44 ^a^	0.37 ^b^	0.04	0.04	0.02	0.99
C16:0	32.81 ^a^	29.29 ^b^	26.04 ^c^	0.59	<0.0001	<0.0001	0.81
C16:1	1.92 ^a^	1.50 ^b^	1.61 ^b^	0.08	0.01	0.01	<0.001
C17:0	0.94 ^a^	0.82 ^b^	0.72 ^c^	0.04	<0.001	<0.001	0.79
C17:1	0.68 ^a^	0.56 ^b^	0.51 ^b^	0.04	0.02	<0.001	0.31
C18:0	19.79 ^c^	22.80 ^b^	23.98 ^a^	0.13	<0.0001	<0.0001	<0.0001
C18:1n9	31.87 ^b^	32.78 ^b^	34.34 ^a^	0.50	<0.001	<0.001	0.49
C18:1*t10*	0.45	0.59	0.68	0.12	0.30	0.14	0.79
C18:1*t11*	0.36	0.41	0.51	0.11	0.44	0.27	0.86
C18:2n6	3.84 ^b^	4.02 ^b^	4.52 ^a^	0.16	<0.001	<0.001	0.32
C18:2*c9;t11*	0.39	0.34	0.32	0.03	0.22	0.09	0.63
C18:3n3	0.44 ^c^	0.67 ^b^	0.78 ^a^	0.02	<0.0001	<0.0001	0.02
C20:0	0.45 ^c^	0.63 ^b^	0.71 ^a^	0.02	<0.0001	<0.0001	0.02
C20:3n6	0.60 ^a^	0.37 ^b^	0.34 ^b^	0.03	<0.0001	<0.0001	<0.001
C20:4n6	0.69 ^b^	0.88 ^a^	0.91 ^a^	0.05	0.01	<0.001	0.08
C20:5n3	0.30 ^b^	0.37 ^a^	0.40 ^a^	0.03	0.02	0.01	0.41
C22:5n3	0.36 ^b^	0.43 ^a^	0.44 ^a^	0.02	0.04	0.01	0.26
C22:6n3	0.14	0.17	0.24	0.06	0.34	0.23	0.70
SFA	57.38 ^a^	56.58 ^a^	54.15 ^b^	0.59	<0.001	<0.001	0.16
MUFA	35.86 ^b^	36.16 ^b^	37.89 ^a^	0.63	0.01	0.02	0.24
PUFA	6.76 ^c^	7.26 ^b^	7.96 ^a^	0.18	<0.001	<0.001	0.55
n3	1.25 ^c^	1.64 ^b^	1.87 ^a^	0.10	<0.001	<0.001	0.39
n6	5.51 ^b^	5.62 ^b^	6.10 ^a^	0.17	0.01	0.01	0.26
n6/n3	4.40 ^a^	3.42 ^b^	3.29 ^b^	0.19	<0.001	<0.001	0.04
UFA/SFA	0.74 ^b^	0.77 ^b^	0.85 ^a^	0.02	<0.001	<0.001	0.16
PUFA/SFA	0.12 ^c^	0.13 ^b^	0.15 ^a^	0.00	<0.0001	<0.0001	0.23
AI	1.04 ^a^	0.91 ^b^	0.77 ^c^	0.03	<0.0001	<0.0001	0.80
TI	2.25 ^a^	2.10 ^b^	1.88 ^c^	0.04	<0.0001	<0.0001	0.35
h/H	1.05 ^c^	1.23 ^b^	1.47 ^a^	0.05	<0.0001	<0.0001	0.53
PI	11.75 ^c^	13.15 ^b^	14.39 ^a^	0.48	<0.001	<0.001	0.87

C14:0 = myristic acid; C14:1 = myristoleic acid; C15:0 = pentadecanoic acid; C16:0 = palmitic acid; C16:1 = palmitoleic acid; C17:0 = eptadecanoic acid; C17:1 = eptadecenoic acid; C18:0 = stearic acid; C18:1n9 = oleic acid; C18:1t10 = trans-10 octadecenoic acid; C18:1 t11 = vaccenic acid; C18:2n6 = linoleic acid; C18:2c9;t11 = conjugated linoleic acid (CLA), isomer c9,t11 (*cis*-9, *trans*-11 octadecadienoic acid); C18:3n3 = alfa-linolenic acid; C20:0 = arachidic acid; C20:3n6 = eicosatrienoic acid; C20:4n6 = eicosatetraenoic acid; C20:5n3 = eicosapentaenoic acid (EPA); C22:5n3 = docosapentaenoic acid (DPA); C22:6n3 = docosahexaenoic acid; SFA = saturated fatty acids; MUFA = monounsaturated fatty acids; PUFA = polyunsaturated fatty acids; UFA/SFA = unsaturated fatty acids/saturated fatty acids ratio; PUFA/SFA = polyunsaturated fatty acids/saturated fatty acids ratio; AI = atherogenic index, TI = thrombogenic index, h/H = hypocholesterolemic/hypercholesterolemic ratio; PI = peroxidation index. ^a–c^ Different superscript letters indicate significant differences between groups at *p* < 0.05.

**Table 7:** Total phenolic content, polyphenols, and antioxidant activity measured in meat (*Longissimus dorsi* muscle) methanolic extracts obtained from Limousin bulls fed olive cake-supplemented diets (expressed as mg phenolic compound/Kg of extract). CTR: control group, receiving conventional concentrate with no olive cake (OC) inclusion; OC10: low-olive cake group, with 10% destoned dried olive cake (DOC) inclusion; OC15: high-olive cake group, with 15% DOC inclusion.

	Group		
Item	CTR	OC10	OC15
DPPH	0.92 ± 0.04 ^a^	2.02 ± 0.1 ^b^	1.8 ± 0.08 ^b^
ABTS	3.6 ± 0.06 ^a^	9.5 ± 0.001 ^b^	9.7 ± 0.04 ^b^
TPC	6.3 ± 0.98 ^a^	23.3 ± 2.59 ^b^	22.4 ± 2.8 ^b^
Hydroxytyrosol	0.80 ± 0.001 ^a^	1.31 ± 0.013 ^b^	2.20 ± 0.04 ^c^
Tyrosol	0.05 ± 0.003 ^a^	0.97 ± 0.064 ^b^	1.46 ± 0.024 ^c^

DPPH = 1,1-diphenyl-2-picrylhydrazyl scavenging activity and ABTS = 2-2′-azino-bis(3-ethylbenzo-thiazoline-6-sulphonate) diammonium salt scavenging activity, expressed as mg TE/Kg extract; TPC = total phenolic content expressed as (mg Ac.Gal Eq/g extract); phenolic characterization, expressed as averages of mg compound/100 g meat ± SD. ^a–c^ Different superscript letters indicate significant differences between groups at *p* < 0.05.

## Data Availability

The data presented in this study are available within the article. Additional data have been previously published in Bionda et al. (2022) [22] https://doi.org/10.3389/fsufs.2022.1077363.
